# Immunohistochemical expression and prognostic value of PD-L1 in Extrapulmonary small cell carcinoma: a single institution experience

**DOI:** 10.1186/s40425-018-0359-1

**Published:** 2018-05-29

**Authors:** Mohammed Salhab, Yazan Migdady, Melanie Donahue, Yiqin Xiong, Karen Dresser, William Walsh, Benjamin J. Chen, James Liebmann

**Affiliations:** 1 Department of Medicine, Hematology and Oncology division, University of Massachusetts Medical School, 55 N lake avenue, Worcester, MA 01655 USA; 2 Pathology Department, Transfusion Medicine, Stanford School of Medicine, Stanford Hospital, 300 Pasteur Drive, Rm H1402, Stanford, CA 94305-5626 USA; 3 Pathology Department, University of Massachusetts Medical School, 55 N lake avenue, Worcester, MA 01655 USA; 4 Lahey Hospital & Medical Center, Hematology and Oncology, Burlington, MA USA

**Keywords:** Extrapulmonary small cell carcinomas, PD-L1 expression and prognostic value, Combined positive score

## Abstract

**Background:**

Extrapulmonary small cell carcinomas (ESCC) are rare but aggressive tumors. Relapses are common despite treatment with chemotherapy and/or radiotherapy. Prospective data for treatment of ESCC are lacking; treatment of these cancers usually incorporates lung small cell carcinoma treatment recommendations. Cancer staging remains the most important prognostic factor. Cancer immunotherapy targeting the PD-1/PD-L1 pathway has shown efficacy in multiple tumor types, and could be an appealing treatment strategy for these rare tumors.

**Methods:**

We investigated PD-L1 expression by immunochemistry (IHC) in ESCCs diagnosed at University of Massachusetts Medical Center, from 1999 to 2016. 34 cases with sufficient material were selected for PD-L1 IHC analysis using clone E1L3N. PD-L1 expression was evaluated using the combined positive score (CPS). Retrospective chart review was performed. We evaluated the incidence and prognostic value of PD-L1 expression in ESCC at our institution.

**Results:**

Twelve out 34 cases (35%) had PD-L1 CPS scores ≥1. Ten cases had CPS scores ranging 1–5, whereas 2 cases had CPS scores > 80. The overall response rate to the standard chemotherapy with/without radiotherapy in the PD-L1 positive group was 80% versus 67% for the PDL-1 negative group (*p*-value 0.67). The median overall survival for the PD-L1 positive group, regardless of stage, was 11.5 months versus 7 months for PD-L1 negative group (*p*-value 0.34). Patients with limited stage disease with positive PD-L1 had a median survival of 53 months compared to 15 months for patients with PD-L1 negative limited stage (*p*-value 0.80).

**Conclusions:**

This study showed that at least one third of our ESCC tissue samples expressed PD-L1. There was a trend for higher response rates to the standard chemotherapy with/without radiotherapy and improved survival in PD-L1 positive patients. Further studies are required to understand the implications of immune dysregulation in these aggressive tumors. PD-L1/PD-1 inhibitors should be investigated in this group of patients.

## Background

Extrapulmonary small cell carcinomas (ESCC) have an aggressive nature characterized by early, widespread metastases. These cancers are rare with a reported incidence of 0.1 to 0.4% in North America and represent up to 5% of all cases of small cell carcinoma [1]. The median survival for limited and extensive disease ranges from 1.4 to 3.5 years and 8 to 12 months, respectively. The overall 5-year survival rate is less than 15% for limited stage patients [1, 2].

Disease stage, either limited or extensive, remains the most important prognostic factor for these cancers [3]. Site of origin of ESCC may also impact prognosis. The most commonly reported primary sites of disease are the gastrointestinal tract, genitourinary system, and ESCC of unknown primary tumor [3, 4].

Most treatment recommendations are derived from small single-institution experiences or extrapolated from pulmonary small cell cancer as prospective trials are lacking for this disease [5, 6]. Multimodality treatment with platinum based chemotherapy with radiation and/or surgery is the most commonly used initial treatment approach for limited stage disease, while platinum based chemotherapy with etoposide is the most widely used regimen for extensive stage patients [5–9]. Despite aggressive initial therapy, most patients with ESCC will relapse with metastatic disease within a year of initial treatment [5, 6].

Cancer immunotherapy targeting the PD-1/PD-L1 pathway is not yet established for ESCC. We evaluated the incidence and the prognostic value of PD-L1 expression in ESCC at our institution. To our knowledge no studies have investigated the value of PD-L1 testing in ESCC.

## Methods

Institutional review board (IRB) approval was obtained. We investigated PD-L1 expression by immunochemistry (IHC) in ESCC diagnosed at University of Massachusetts Medical Center between 1999 and 2016. Forty-five patients with ESCC were identified. From these patients, 34 had specimens with sufficient material for PD-L1 IHC analysis. Specimens included cytology cell blocks, needle core biopsies, and whole tissue sections from resection specimens.

Patients included for analysis had a pathologic diagnosis of ESCC and no evidence of lung disease based on chest CT scan at time of diagnosis. Pathologic diagnosis included morphology consistent with small cell carcinoma, and/or positive immunohistochemical staining with synaptophysin, chromogranin, and/or CD56. Well-differentiated neuroendocrine tumors, including carcinoid tumors, were excluded. Disease stage was classified as extensive (ED) or limited (LD), using the Veterans’ Affairs Lung Study Group criteria, by reviewing radiologic records [10]. LD was defined as tumor confined to the primary site and regional lymph nodes. ED was defined as tumor extending beyond loco-regional boundaries. Chart reviews were conducted to assess treatment modalities, age at diagnosis, staging, site of origin, response to treatment, and survival. Response to treatment was defined as at least partial response, based on RECIST (Response Evaluation Criteria In Solid Tumors) criteria [11].

Immunohistochemical studies were performed on 5-um sections of formalin-fixed, paraffin-embedded tissue. Slides were first deparaffinized, and rehydrated. Antigen retrieval was carried out with 0.01 M citrate buffer at pH 6.0. Slides were heated in a 770-W microwave oven for 14 min, cooled to room temperature, and rinsed in distilled water before staining. All the slides were stained on the Dako Autostainer (Dako Corporation, Carpinteria, CA). The sections were first blocked for endogenous peroxidase activity with an application of Dual Endogenous Block (Dako) for 10 min, followed by a brief buffer wash. The slides were then incubated with primary antibody for 30 min. The rabbit monoclonal antibody to PD-L1 (clone E1L3N, Cell Signaling, #13684) was used at a dilution of 1:500, and diluted with Dako Antibody Diluent (Dako). Following a buffer rinse, sections were incubated with Ultraview Detection (Ventana Medical Systems, Tuscon, AZ) for 30 min. The sections were washed, and treated with a solution of diaminobenzidine and hydrogen peroxide (Dako) for 10 min, to produce the visible brown pigment. After rinsing, a toning solution (DAB Enhancer, Dako) was used for 2 min to enrich the color. Following rinsing, the sections were counterstained with hematoxylin, dehydrated, and cover slipped with permanent media. Sections of tonsil, placenta, and classical Hodgkin lymphoma tissue with known positivity for PD-L1 were used as positive controls for staining.

PD-L1 IHC scoring was performed using the combined positive score (CPS), previously described for the scoring of PD-L1 in esophageal adenocarcinoma [12, 13]. CPS is defined as the total number of PD-L1 positive cells (tumor, lymphocytes, and macrophages) divided by the total number of tumor cells. The average CPS of 2 hotspots in each sample was recorded. Two pathologists independently reviewed the IHC studies with agreement in most cases (> 80%), followed by discussion to arrive at a consensus in the remaining cases. CPS ≥1 was considered positive.

### Statistical analysis

All survival and subgroup analyses were performed using GraphPad Prism software, with curve comparisons performed using log-rank (Mantel-Cox) tests. Kaplan-Meier survival curves were generated using GraphPad Prism software. Response rates to treatment were compared using Fisher test with *p* > 0.05 accepted as statistically significant.

## Results

Demographic characteristics of the patients and the site of origin of their cancers are shown in Table 1. The most common confirmed primary sites were from the genitourinary tract. However, cancers of unknown primary origin constituted the second most common group.

**Table 1:** Baseline characteristics of ESCC patients

Characteristics	Number (%)
Total patients (n)	34
Age	
Median	68
Range	20–86
Sex	
Male	19 (56%)
Female	15 (44%)
Stage	
Limited	11 (32%)
Extensive	23 (68%)
Localization	
Genitourinary	18 (53%)
Unknown primary	10 (29%)
Gastrointestinal	5 (15%)
Other (Palate)	1 (3%)

**Fig. 1:**
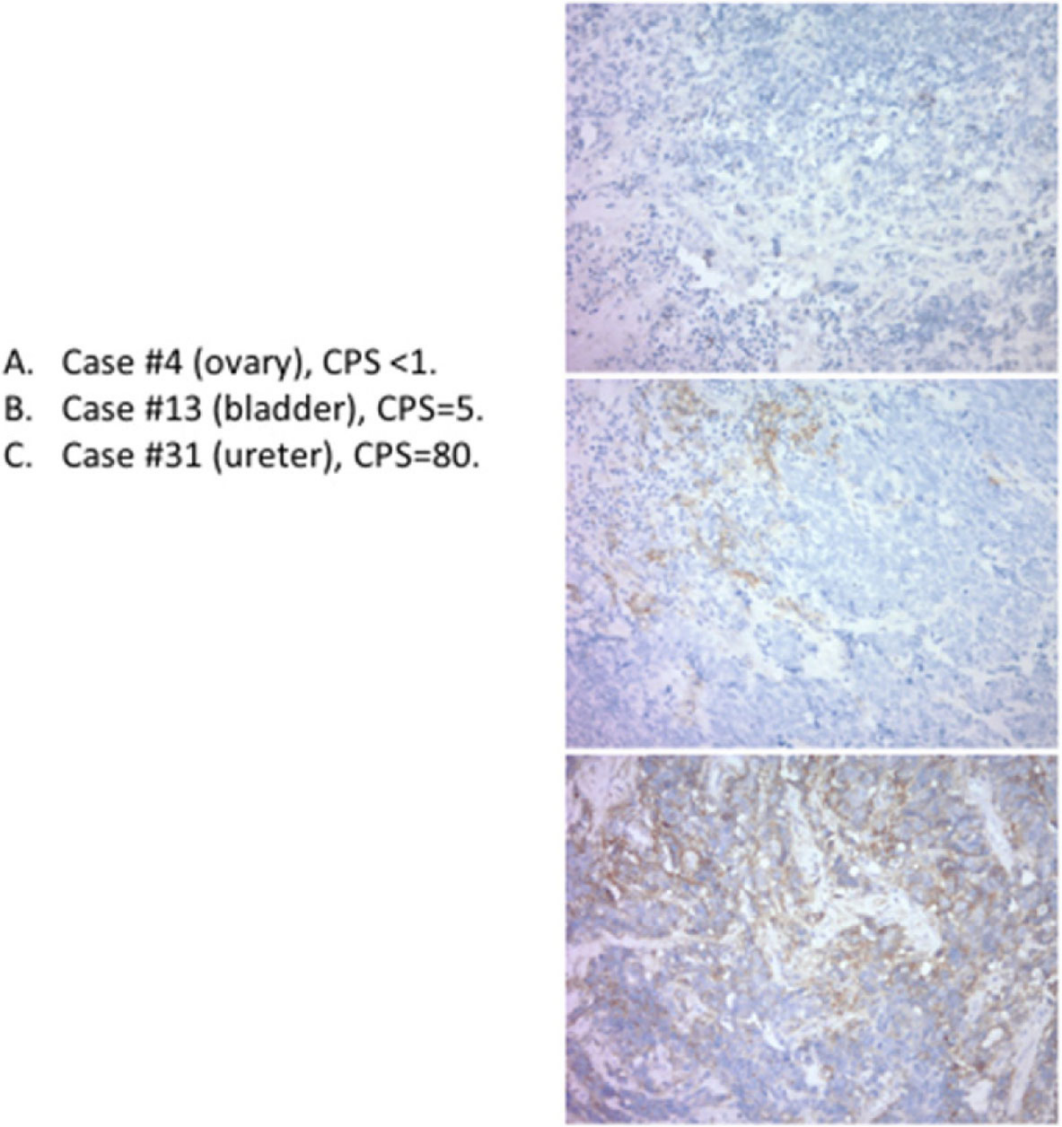
Examples of extrapulmonary small cell carcinoma stained for PD-L1 expression graded by combined positive score

Twelve specimens (35%) showed positive PD-L1 expression (CPS ≥1, Fig. 1). Of the positive specimens, 2 showed diffuse PD-L1 expression in both tumor and stromal cells with CPS > 80. One of these cases arose from the ureter and the other from the gall bladder. The other 10 specimens that were considered positive had CPS scores of 1–5, primarily due to focal positive staining of non-tumor immune cells adjacent to the tumor. Twenty-two cases (65%) were negative for PD-L1 expression (CPS < 1).

**Fig. 2:**
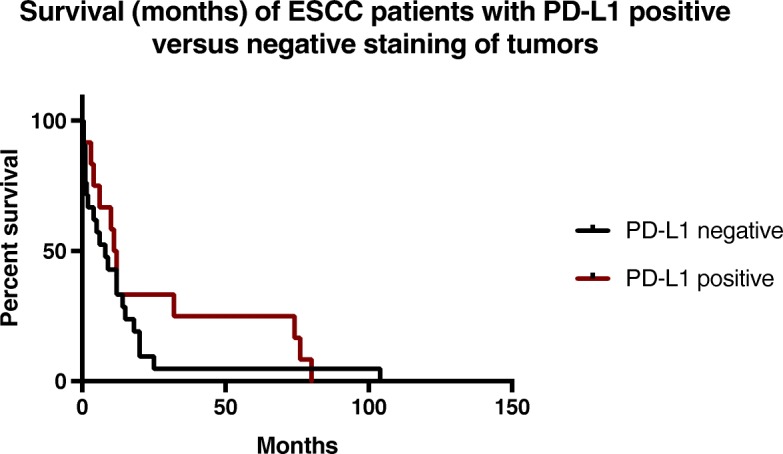
Overall survival (months) of ESCC patients in PD-L1 positive versus negative groups (p 0.34)

All patient with limited stage received a bimodality treatment with chemoradiation. Fifty-six percent of extensive stage patients received chemotherapy. One patient received platinum and irinotecan, while all other patients with extensive disease who received chemotherapy were treated with platinum and etoposide. The other 46% of patients with extensive stage disease received only supportive care with no chemotherapy; 15% of those patients declined chemotherapy, 9% expired before obtaining the final pathology read but received supportive care while in the intensive care unit, and the remainder had very poor performance status with advanced age. Fifteen percent patients had surgical resection of their primary cancer, in the setting of a diagnostic evaluation. The overall response rate in the PD-L1 positive group was 80% versus 67% in the PD-L1 negative group (*p*-value 0.67) [Table 2]. The median overall survival for the PD-L1 positive group, regardless of stage, was 11.5 months versus 7 months for the PD-L1 negative group (*p*-value 0.34) [Fig. 2]. Patients with limited stage disease with positive PD-L1 staining had a median 53 months overall survival compared to PD-L1 negative patients with limited disease whose median overall survival was 15 months (*p*-value 0.80) [Table 3]. Extensive stage patients had median overall survival that was similar between PD-L1 positive and negative groups, 4 months vs 5 months respectively (*p*-value 0.50).

**Table 2:** The overall response rate and median survival between PD-L1 positive and negative groups across all cancer stages

	PD-L1 positive (*n* = 12)	PD-L1 negative (*n* = 22)	*P* value
Received treatment	10 (83%)	16 (73%)	
Response rates to treatment	8 (80%)	11 (67%)	0.67
Median overall survival (mo.)	11.5	7	0.34

**Table 3:** The overall response rate and median survival between limited stage PD-L1 positive and negative groups

	Limited stage disease		*P* value
	PD-L1 positive	PD-L1 negative	
	(*n* = 6)	(*n* = 5)	
Male sex (%)	4 (66%)	2 (40%)	
Received treatment	6 (100%)	5 (100%)	
Response rates to treatment	6 (100%)	5 (100%)	
Alive at 1 year	6 (100%)	4 (80%)	
Median overall survival (mo.)	53	15	0.80

Of the 25 patients who relapsed, 14 patients relapsed after 2007 when the FDA approved Topotecan for relapsed small cell carcinoma of the lung. Two Patients declined additional chemotherapy, two patients received Topotecan without any response and the remaining 10 patients progressed rapidly and didn’t get treated due to poor performance status. None of the patients received second line chemotherapy before 2007.

## Discussion

Our data showed that 35% of ESCC tumor samples were positive for PD-L1(CPS ≥1). PD-L1 positive tumors showed a trend toward improved median overall survival and there was separation of survival curves between the PD-L1 positive and negative groups at around 1 year, though not statistically significant.

Treatment of ESCC tends to follow the treatment plan utilized for small cell lung cancer. First line treatment with platinum-based chemotherapy remains the standard. Second line treatments are lacking for all patients with small cell carcinomas, regardless of tissue of origin, with topotecan as the only FDA approved drug in the second line setting [14]. Immunotherapy is a promising treatment for small cell carcinomas. PD-L1 inhibitors have been investigated in small cell lung cancer in a phase IB study (NCT02054806) as second line therapy. In that trial, 20 patients with SCLC expressing PD-L1 by immunohistochemistry were treated with pembrolizumab. Objective responses were observed in seven cases (35%) [15]. Both nivolumab and ipilimumab have also demonstrated activity in early-phase clinical studies. In a phase II study, 216 patients were assigned to treatment with nivolumab or nivolumab plus ipilimumab at three different dose combinations. An objective response was achieved in 10% of patients receiving nivolumab only versus 21% of patients receiving both nivolumab and ipilimumab at any dose combination [16].

PD-1/PD-L1 inhibition has shown efficacy in a variety of malignancies in both first line and subsequent therapy [17–19]. Many trials have attempted to identify subsets of patients who are most likely to benefit from PD-L1 checkpoint inhibition by assessing PD-L1 expression in tumor specimens and/or tumor microenvironment. Cancers with high PD-L1 expression have consistently demonstrated a higher response rate to PD-1/PD-L1 antibodies than cancers that lack PD-L1 expression, although the use of such inhibitors are not limited to the positive expressers. The use and pitfalls of PD-L1 assays have been reviewed recently [20]. As it remains controversial regarding which scoring system is preferable to grade the expression, we used the CPS because of the recent trend in using this scoring in other tumors mainly gastrointestinal [12, 13]. We aimed to provide more information about the tumor cells and their microenvironment (lymphocytes, and macrophages), for this unstudied area of such a rare disease.

Tumor PD-L1 status may provide prognostic or predictive information. A recently published clinical trial conducted in patients with advanced non-squamous, non-small-cell lung cancer, demonstrated a higher response rate to chemotherapy in patients whose tumors had high expression of PD-L1 compared to patients whose tumors failed to express PD-L1 [21, 22]. Similarly, in our study, ESCC with PD-L1 expression showed higher response rates to chemotherapy than ESCC that lacked PD-L1 expression. Additionally, in our study patients whose tumors tested positive for PD-L1 had improved median overall survival compared with patients whose tumors did not express PD-L1. The lack of statistical significance in differences of overall survival likely reflects the small sample size in this single institution review of patients with a rare malignancy. While disease staging and the site of origin of ESCC remain the most important prognostic factors, these results suggest that PD-L1 expression could be a predictive variable for prognosis and response to chemotherapy in ESCC.

The proposed rational of improved responses in patients with PD-L1 expression could be related to the presence of lymphocytes at the microenvironment of the tumor cells which could be of help to initiate apoptosis and inflammatory reaction, thus enhancing tumor response to chemotherapy and radiation. It is possible that the enhanced cytotoxicity of combination therapy could be due to the release of tumor neoantigens from cells killed by chemotherapy. Combining immunotherapy with chemotherapy as an upfront treatment is of interest at this point for all small cell carcinomas as chemotherapy showed the potential to increase the PD-L1 expression within the tumor microenvironment, which will enhance the responses to PD-1 inhibitors [23–25].

## Conclusion

In conclusion, this study demonstrates the incidence of PD-L1 expression in a small cohort of ESCC, and suggests that PD-L1 expression in ESCC may be of prognostic and predictive relevance. Further studies are required to understand the implications of immune dysregulation in these aggressive tumors. PD-L1/PD-1 inhibitors should be investigated in this group of patients given the positive expression of PD-L1 in one third of patients in our review.

## Abbreviations

CPS: Combined positive score
ED: Extensive
ESCC: Extrapulmonary small cell carcinomas
IHC: Immunochemistry
LD: Limited
RECIST: Response evaluation criteria in solid tumors
